# Electric field effects of pulsed field ablation through adjacent non-cardiac tissues in an *in vitro* hybrid model

**DOI:** 10.3389/fcvm.2026.1811901

**Published:** 2026-08-04

**Authors:** Denis Fedorov, Laura Dippel, Jan Minners, Johannes Steinfurt, Julian Mueller, Dirk Westermann, Heiko Lehrmann, Thomas Arentz, Martin Eichenlaub

**Affiliations:** Department of Cardiology and Angiology, University Heart Center Freiburg/Bad Krozingen, Medical Center, University of Freiburg, Bad Krozingen, Germany

**Keywords:** atrial arrhythmias, electroporation, esophagus, pulsed field ablation, vegetable model

## Abstract

**Background:**

Endocardial ablation of atrial arrhythmias may occasionally produce insufficient lesion depth. Pulsed-field ablation (PFA) may generate measurable electroporation effects beyond interposed non-cardiac tissues, but field propagation across such tissues remains poorly understood.

**Methods:**

We introduced an *in vitro* model to visualize electroporation in potato slices overlaid by *ex vivo* porcine tissues, including esophagus, aortic root, and pulmonary trunk. A control group received PFA without any barrier. Lesions were created using a commercially available multielectrode PFA catheter across three subsets using different numbers of applications. Depth of irreversible electroporation, total lesion depth (irreversible and reversible electroporation), and irreversible electroporation surface area were quantified.

**Results:**

Across 60 lesions, two-way ANOVA showed significant effects of barrier tissue and number of applications on all primary endpoints (all *p* < 0.001). A significant tissue-by-dose interaction was observed only for total lesion depth (*p* < 0.001). Compared with control, irreversible electroporation depth was lower beneath the esophageal, aortic root, and pulmonary trunk by 2.01, 2.21, and 1.44 mm, respectively. Total lesion depth was unchanged beneath the esophagus but lower beneath the aorta and pulmonary trunk. Irreversible electroporation area was greater beneath the esophagus, lower beneath the aorta, and unchanged beneath the pulmonary trunk.

**Conclusion:**

Our findings show that measurable electroporation effects can be produced in a hybrid potato model beyond *ex vivo* non-cardiac tissues, with tissue-dependent differences in lesion depth and lesion area.

## Introduction

Catheter ablation represents a cornerstone of rhythm control therapy (1). Among emerging technologies, pulsed field ablation (PFA) has gained growing attention as a novel energy source offering a high efficacy and safety profile. Unlike thermal ablation modalities such as radiofrequency or cryoablation, PFA induces cell death primarily by pore formation in lipid cell membrane when pulsed electric fields interact with biological tissue (2). Because different cell types have distinct electroporation thresholds (3), PFA provides a certain tissue selectivity with myocardial targeting while potentially sparing adjacent non-cardiac structures, including esophagus, aorta, veins, lungs, and nerves (4–6). In clinical practice, myocardial PFA is typically performed for pulmonary vein isolation in patients undergoing first ablation procedure (7). However, especially in patients with persistent atrial fibrillation or atypical atrial flutter, a pulmonary vein isolation-only approach is often insufficient, and additional substrate modification is required. The left atrial posterior wall (LAPW), which shares its embryological origin with the pulmonary veins, is a recognized source of atrial fibrillation triggers beyond the pulmonary veins (8, 9). Ablation of the LAPW has been shown to significantly reduce AF burden, yet achieving durable isolation frequently requires a hybrid approach (10). PFA has also been employed for LAPW isolation and has demonstrated durable success in about 85% of cases (11). Moreover, extra-pulmonary vein triggers, macro-reentrant circuits, and focal atrial tachycardias can originate from regions adjacent to major blood vessels, such as Bachmann's bundle near the pulmonary artery or the mitral isthmus adjacent to the coronary sinus, representing areas that are often difficult to achieve durable ablation using a purely endocardial approach.

In our experiment, we used an *in vitro* hybrid model to evaluate whether *ex vivo* non-cardiac tissues modify measurable electroporation effects in an underlying potato phantom and to compare tissue-specific attenuation.

## Materials and methods

A potato-based model was used in the present study, which has been shown to provide a convenient and cheap alternative to animal models for visualization and quantification of PFA lesions (12–14). Firm potatoes (table variety) were purchased from a local supermarket. One porcine esophagus, one aortic root with ascending aorta, and one pulmonary trunk were obtained from a single animal commercially slaughtered for food production. These tissues were by-products, and no animal was sacrificed specifically for this study.

### Experimental setup

Pulsed field energy was delivered using the Farapulse Pulsed Field Ablation System with a 31-mm pentaspline catheter (Farawave) and the Farastar generator (Boston Scientific, Marlborough, MA, USA). Potatoes were cut transversely into slices and placed in a container filled with half-normal saline (0.45% NaCl) maintained at 37 °C. The porcine esophagus, aortic root with ascending aorta, and pulmonary trunk were opened by a single longitudinal incision to create flat tissue layers.

In the control group (C group), the PFA catheter was positioned directly on the potato slices. In the barrier groups, a single layer of non-viable porcine esophagus (E group), aortic root/ascending aorta (A group), or pulmonary trunk (*P* group), respectively, was interposed between the potato slice and the catheter.

### PFA delivery protocol

Ablation was performed with the catheter in the “flower” configuration in bipolar mode at 2,000 V per application. After each set of applications, the catheter was rotated by approximately 35° by visual estimation and the applications were repeated. Each group (C, E, A, P) was divided into three subsets according to the number of applications at each position:

- 2 + 2: two applications at the initial orientation and two after rotation
- 4 + 4: four applications at the initial orientation and four after rotation
- 6 + 6: six applications at the initial orientation and six after rotation

Each subset included *n* = 5 slices.

Because the pentaspline “flower” has a subtly nonplanar surface (slight spline twist about the frontal axis) and a distal tip that limits full contact on a rigid surface, we placed an additional potato slice on top of the catheter (“sandwich”-like stabilization; Supplementary Figure 1). Manual pressure was applied through the upper slice to stabilize the catheter and improve electrode-tissue contact. Due to the limited dimensions of the barrier tissues, applications within each specimen were delivered through the same or a closely adjacent region.

### Lesion visualization and image analysis

Slices were exposed in a 0.5% 2,3,5-triphenyltetrazolium chloride (TTC) solution for approximately 12 h for lesion visualization (12). Areas of presumed irreversible (TTC-negative, pale) and reversible electroporation (TTC-positive, red) were distinguished as described previously (12).

The catheter footprint area in the “flower” configuration was estimated by tracing the catheter outline on paper, rotating it by ∼35°, and tracing again to capture the combined projected area (Supplementary Figure 2).

Image analyses were performed in Fiji software (RRID:SCR_002285) (15). The following parameters were obtained: maximal depth of irreversible electroporation and total lesion depth (irreversible+reversible electroporation), irreversible electroporation surface area, and the PFA catheter footprint area. Lesion and footprint areas were segmented using the Trainable Weka Segmentation plugin.

Barrier tissue thickness and lesion dimensions were estimated from calibrated images acquired with a ruler positioned next to the barrier tissues or potato slices. Because the tissue edges and ruler were not always perfectly aligned with the imaging plane, barrier tissue thickness measurements were considered approximate.

### Regulatory statement

The study draft was reviewed by the competent veterinary authority (Fachbereich Veterinärwesen und Lebensmittelüberwachung/Tiergesundheit und Tierschutz, Landratsamt Breisgau-Hochschwarzwald, Germany), which confirmed that no additional approval or registration was required for this one-time *in vitro* experiment.

### Statistical analysis

Continuous variables are reported as mean ± SD [min-max]. Interposed tissue type and number of PFA applications were entered as fixed factors. Two-way analysis of variance (ANOVA) was performed for each primary endpoint, including interposed tissue type, number of applications, and their interaction. Given that each interposed tissue type was represented by a single *ex vivo* preparation, inferential analyses were considered exploratory and were performed at the lesion level. *post hoc* comparisons between each interposed tissue group and the control group were performed using Dunnett's adjustment for multiple comparisons. Effect sizes are reported as partial eta-squared (partial *η*²). Ninety-five percent confidence intervals are provided for Dunnett-adjusted comparisons with the control group. A *p*-value < 0.05 was considered statistically significant. Analyses were performed in SPSS v30 (IBM, New York, NY, USA).

## Results

**Figure 1:**
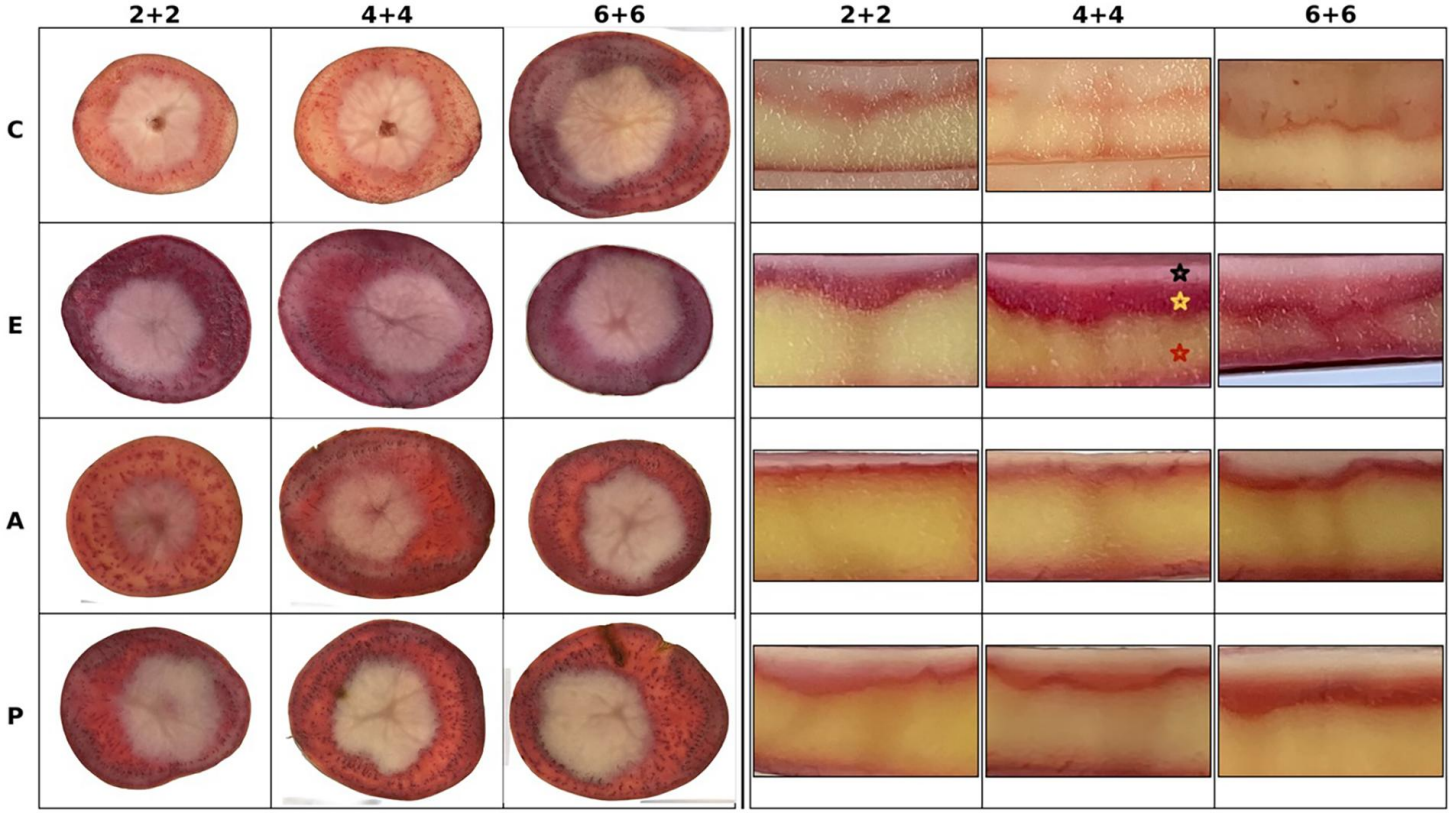
Irreversible electroporation area and lesion depth. Groups (rows) are labeled as: C – control; E – esophagus; A – aortic root; P – pulmonary trunk. Subsets (columns) are labeled according to the number of applications. Images are not shown at the same scale. Black star – irreversible electroporation (pale area), yellow star – reversible electroporation (red area), red star – non-electroporated tissue.

**Figure 2:**
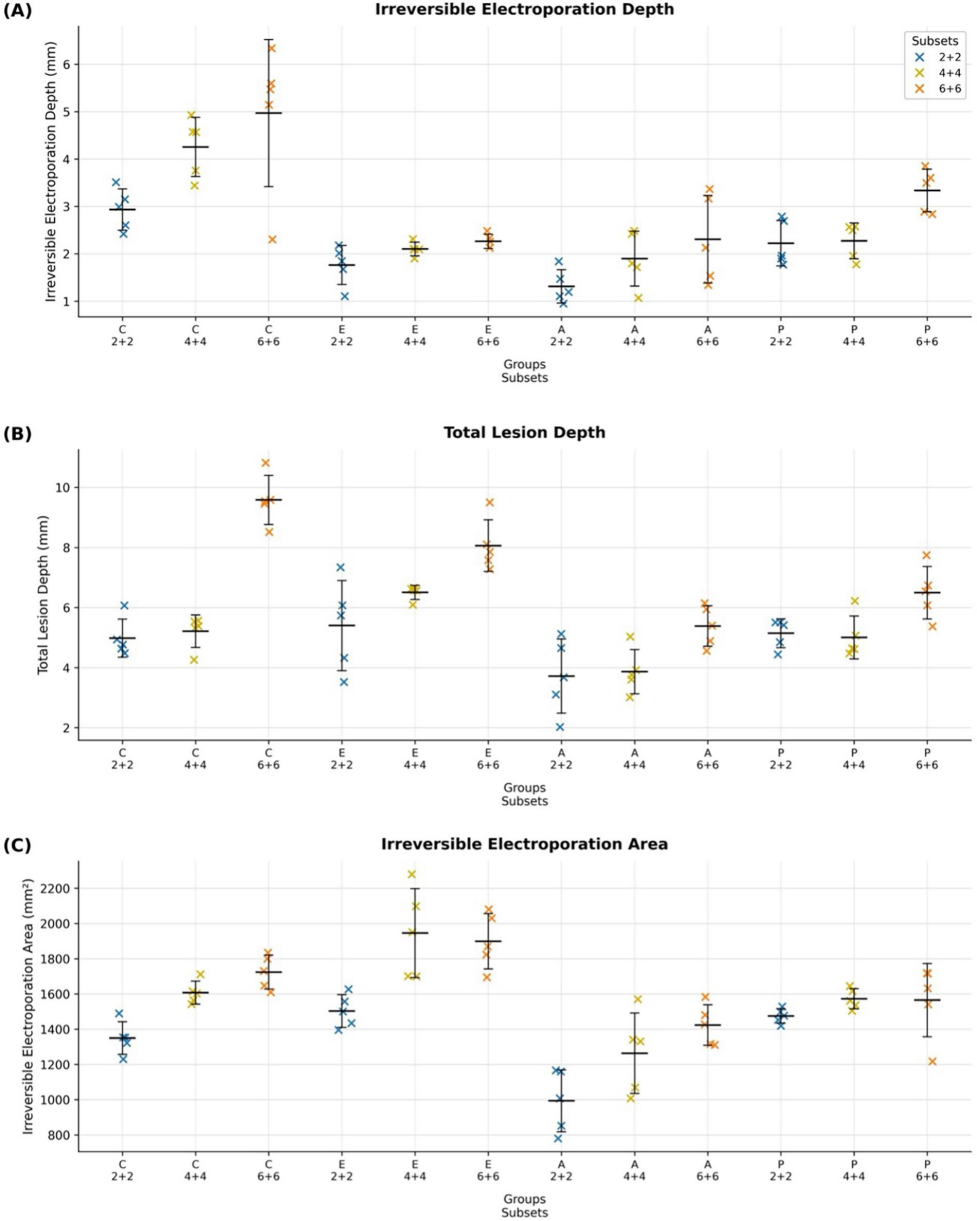
Lesion measurements across groups and subsets. **(A)** (top): Irreversible Electroporation Depth (mm), **(B)** (middle): Total Lesion Depth (mm), and **(C)** (bottom): Irreversible Electroporation Area (mm^2^). Each x-shaped marker represents an individual slice. Thick horizontal black bars represent the mean. Thin vertical black bars represent standard deviation. Groups are labeled as: C – control; E – esophagus; A – aortic root; P – pulmonary trunk. Subsets are labeled according to the number of applications.

A total of 60 lesions were analyzed (4 groups×3 subsets×5 slices). All slices showed both TTC-negative (irreversible) and TTC-positive (reversible) electroporation (Figure 1). Summary lesion parameters for all 60 slices are shown in Figure 2. Lesion characteristics of the different tissue groups are presented in Table 1.

**Table 1:** Lesion characteristics of the different tissue groups (pooled across subsets).

Group	*n*	Irreversible electroporation depth, mm	Total lesion depth, mm	Irreversible electroporation area, mm^2^
C	15	4.05 ± 1.27 [2.30–6.33]	6.59 ± 2.28 [4.26–10.81]	1,560.48 ± 180.31 [1,230.36–1,834.31]
E	15	2.04 ± 0.33 [1.11–2.48]	6.65 ± 1.47 [3.52–9.50]	1,782.81 ± 264.44 [1,396.12–2,279.12]
A	15	1.84 ± 0.74 [0.95–3.37]	4.32 ± 1.15 [2.02–6.15]	1,226.82 ± 247.43 [780.07–1,583.28]
P	15	2.61 ± 0.67 [1.77–3.85]	5.55 ± 0.96 [4.44–7.74]	1,537.69 ± 125.95 [1,217.18–1,718.30]

All data are shown as mean ± standard deviation [min-max]. Groups are labeled as: A – Aortic root; C – control; E – esophagus; P – pulmonary trunk.

In two-way ANOVA models, both barrier tissue type and number of applications had significant main effects on irreversible electroporation depth, total lesion depth, and electroporation area (all *p* < 0.001; Table 2). The barrier tissue-by-dose interaction was significant for total lesion depth, F(6,48) = 5.29, *p* < 0.001, partial *η*² = 0.398, but not for irreversible electroporation depth or area.

**Table 2:** Two-way ANOVA results and partial *η*².

Endpoint	Effect	F statistic	*p*-value	Partial *η*²
Irreversible electroporation depth	Tissue barrier type	F(3,48) = 35.26	<0.001	0.688
	Dose subset	F(2,48) = 15.87	<0.001	0.398
	Tissue-by-dose	F(6,48) = 1.68	0.147	0.174
Total lesion depth	Tissue barrier type	F(3,48) = 25.61	<0.001	0.615
	Dose subset	F(2,48) = 55.52	<0.001	0.698
	Tissue-by-dose	F(6,48) = 5.29	<0.001	0.398
Irreversible electroporation area	Tissue barrier type	F(3,48) = 35.75	<0.001	0.691
	Dose subset	F(2,48) = 27.14	<0.001	0.531
	Tissue-by-dose	F(6,48) = 2.06	0.076	0.205

Compared with control, irreversible electroporation depth was lower after the PFA application through the esophagus by 2.01 mm (Dunnett-adjusted 95% CI −2.59 to −1.43 mm), through the aorta by 2.21 mm (95% CI −2.79 to −1.64 mm), and through the pulmonary trunk by 1.44 mm (95% CI −2.02 to −0.86 mm). Irreversible electroporation area was larger after the PFA application through the esophagus by 222.33 mm^2^ (95% CI +91.18 to +353.49 mm^2^), smaller through the aorta by 333.66 mm^2^ (95% CI −464.82 to −202.50 mm^2^), and numerically smaller but not significantly different through the pulmonary trunk by 22.78 mm^2^ (95% CI −153.94 to +108.38 mm^2^; Table 3).

**Table 3:** Mean differences versus control with 95% confidence intervals.

Endpoint	Comparison	Mean difference	Dunnett-adjusted 95% CI	Adjusted *p*-value
Irreversible electroporation depth, mm	Esophagus vs. control	−2.01	−2.59–−1.43	<0.001
	Aortic root vs. control	−2.21	−2.79–−1.64	<0.001
	Pulmonary trunk vs. control	−1.44	−2.02–−0.86	<0.001
Total lesion depth, mm	Esophagus vs. control	+0.06	−0.68–+0.81	0.994
	Aortic root vs. control	−2.27	−3.01–−1.53	<0.001
	Pulmonary trunk vs. control	−1.04	−1.79–−0.30	0.004
Irreversible electroporation area, mm^2^	Esophagus vs. control	+222.33	+91.18–+353.49	<0.001
	Aortic root vs. control	−333.66	−464.82–−202.50	<0.001
	Pulmonary trunk vs. control	−22.78	−153.94–+108.38	0.952

Compared to the estimated Farawave 31-mm catheter footprint in the “flower” configuration (807.3 mm^2^; Supplementary Figure 2), the mean irreversible electroporation areas exceeded this footprint in all groups: control group 1,560.5 mm^2^ (193% of footprint), aortic root 1,226.8 mm^2^ (152%), esophageal 1,782.8 mm^2^ (221%), and pulmonary trunk 1,537.7 mm^2^ (190%).

### Barrier tissue observations

Measured thicknesses of the barrier tissues were: retrocardiac esophagus 2.7–4.0 mm, aortic root/ascending aorta approximately 2.5 mm, and pulmonary trunk approximately 2.3 mm. Macroscopically, small white spots were observed on the esophageal mucosa after PFA applications, a reddish ring at the pulmonary trunk contact site, and no visible changes in the aortic root (Supplementary Figures 3–5). Because the esophageal mucosa was not systematically inspected after each application, the application number at which the white spots first appeared could not be determined.

## Discussion

In this *in vitro* hybrid potato model, we demonstrated that PFA delivered through tissues adjacent to the heart (esophagus, aortic root, and pulmonary trunk) can induce both reversible and irreversible electroporation in the potato tissue. All barrier tissues reduced irreversible electroporation depth compared with direct application, with the greatest attenuation observed for the aortic wall, followed by the esophagus and the pulmonary trunk. Delivery through the esophagus resulted in a larger irreversible electroporation area than direct application, suggesting tissue-dependent differences in field distribution. Increasing the number of applications showed a dose-response, with progressive increases in total electroporation depth and irreversible electroporation area.

### Esophagus

The esophagus runs along the LAPW, a recognized source of atrial fibrillation triggers beyond the pulmonary veins. Endocardial and epicardial ablation of the LAPW in addition to pulmonary vein isolation has been evaluated and, in some studies, associated with reduced arrhythmia recurrence in patients with atrial fibrillation (10, 11, 16). Distance between esophagus and LAPW is about 3.3–13.5 mm (17). The retrocardiac esophageal wall thickness is 2.5 ± 1.0 mm (range 1.5–4.5 mm) at autopsy (17), and approximately 1.94 mm (95% CI 1.77–2.09 mm) on computed tomography (CT) in dilated segments, aligning well with measurements of the animal esophagus used in the present study (18). LAPW thickness is non-uniform, with the superior portion thinnest. CT-based LAPW thickness estimates 1.48 ± 0.21 mm (19), while autopsy measures distance from endocardium to epicardium 2.2–6.5 mm (range 1.1–12.0 mm) (17). In our model, irreversible electroporation depth through the esophagus was reduced compared with control, total lesion depth was similar, and irreversible electroporation area was larger. These findings show that electric field effects can be detected beyond an interposed single layer of the esophagus in this simplified hybrid model.

Thermal ablation on the LAPW carries a risk of collateral esophageal injury, including the rare but potentially lethal complication of atrio-esophageal fistula. In contrast, early preclinical data suggest distinct esophageal responses to PFA. Direct intraluminal and extraluminal PFA on the esophagus and PFA within atria and large veins proximal to the esophagus produced mucosal lesions in several *in vitro* animal models. Mucosa normalization was observed by day 7 and no esophageal lesions were reported at 14–30 days except for the direct extraluminal application using a monophasic external defibrillator, which led to a formation of a visible focal external muscular scar (4–6, 20–24). In our model, white mucosal spots were observed where the esophageal mucosa directly contacted the electrodes. Because no histological examination or temperature monitoring was performed, the mechanism of these macroscopic changes cannot be identified.

This experimental model should not be viewed as a direct simulation of clinical atrial PFA. In standard AF ablation, energy is delivered endocardially, whereas the esophagus is one of the adjacent structures at risk of collateral damage. Our hybrid model demonstrates pulsed field propagation across a flattened, *ex vivo* single layer of an esophagus in simplified *in vitro* conditions, but it does not confirm the feasibility, efficacy, or safety of transesophageal PFA delivery to myocardial targets.

### Aorta and pulmonary trunk

The sinuses of Valsalva of the aortic root provide access to ventricular arrhythmias arising from the aortic cusps (25). The non-coronary cusp has also been used for radiofrequency ablation of focal atrial arrhythmias originating near the His bundle (26) and the atrioventricular node (27), and select anteroseptal accessory pathways can be approached from this region (28).

Both right and left pulmonary arteries course above the left atrial roof and Bachmann's bundle, allowing signal recording and targeted stimulation from within the vessel lumen (29). While Bachmann's bundle, an epicardial structure, is a key part of interatrial conduction, it may also be a source of focal atrial tachycardia, atrial fibrillation triggers, or participate in macro-reentrant circuits. A nonrandomized study in 60 patients with long-standing persistent atrial fibrillation reported that adding an epicardial Bachmann's bundle ablation to a hybrid epicardial-endocardial pulmonary vein and LAPW isolation increased freedom from any atrial arrhythmia recurrence at 1-year follow-up (30).

Several case reports have described successful radiofrequency ablation of Bachmann's bundle from the right pulmonary artery (31, 32). However, lesion formation can be limited by high local coronary flow, adjacent to Bachmann's bundle. To overcome this limitation, Kurotobi et al. employed rapid ventricular pacing to transiently reduce coronary flow in the sinoatrial nodal artery of the left circumflex artery and permit sufficient conductive heating to reach Bachmann's bundle (33). Because PFA lesion formation is less dependent on conductive heating than radiofrequency ablation, it may be less sensitive to perfusion-related cooling. However, our model cannot predict whether this would lead to effective myocardial lesion formation *in vivo*.

The structural and hemodynamic differences between the aorta and the pulmonary trunk increase both thickness and stiffness of the aortic wall and therefore potentially contributing to greater attenuation of electric field penetration through the aortic wall compared with the pulmonary trunk. This is consistent with our findings that delivery across the aorta produced the shallowest irreversible electroporation depth and the smallest irreversible electroporation area among the tested barrier tissues.

The observed differences between the aortic root and pulmonary trunk should be interpreted cautiously. Similar to the esophageal tissue, the aortic root and the pulmonary trunk were opened and flattened and therefore did not reproduce native vessel geometry, intraluminal blood flow, pulsatility, pressure, or dynamic tissue conditions.

In this simplified *in vitro* model, measurable electroporation effects were observed in a potato phantom beyond selected *ex vivo* porcine tissues. The barrier tissues modified lesion depth and area in a tissue-specific manner. The findings support further investigation of electric field propagation across adjacent non-cardiac tissues using computational modeling and *in vivo* validation, but they do not confirm myocardial lesion formation, transmurality, durability, or clinical safety.

### Limitations

The proposed model is anatomically simplified. The esophagus was opened longitudinally and flattened, and therefore did not reproduce tubular anatomy, luminal environment, wall tension, perfusion, or the relationship with surrounding tissues. Our model did not include interposed epicardial fat pads or parietal pericardium between the LAPW and the esophagus (17). These structures may substantially alter electric field distribution and lesion formation *in vivo*. Only one esophagus, one aortic root with ascending aorta, and one pulmonary trunk were used across all application subsets, therefore variability between specimens could not be assessed. The potato phantom may overestimate lesion size relative to animal myocardium (34). The experiment was conducted in a saline bath, which does not reflect the intraluminal environment of the esophagus. Tissue thickness was estimated from calibrated images rather than measured directly at each catheter contact site. Possible misalignment between the tissue, ruler, and imaging plane may have reduced measurement precision. Therefore, tissue thickness was reported descriptively and was not included as a covariate in the statistical analysis. Because the same or closely adjacent region of each barrier specimen was used for multiple applications, prior PFA may have altered the tissue properties and influenced subsequent lesion formation. The conductivity of the saline bath and the electrical properties of the interposed tissues were not measured. Energy was delivered in bipolar mode at a fixed 2,000 V; unipolar delivery and lower voltages were not evaluated. Placing the pentaspline catheter between two potato slices may have affected lesion size and/or depth, moreover the stabilizing pressure applied through the upper slice was not measured or standardized; differences in contact pressure may have influenced catheter-tissue contact and therefore lesion depth and lesion area. Catheter rotation by approximately 35° was estimated visually; small deviation in rotation may have affected lesion depth and lesion area. The barrier tissues were assessed macroscopically only; microscopic evaluation and temperature monitoring should be included in future *in vivo* experiments.

## Data Availability

The original contributions presented in the study are included in the article/Supplementary Material, further inquiries can be directed to the corresponding author.
