# Supplementary material for: Electric field effects of pulsed field ablation through adjacent non-cardiac tissues in an in vitro hybrid model
Source: Front Cardiovasc Med. 2026 Aug 4;13:1811901. doi: 10.3389/fcvm.2026.1811901 (PMC13481181; doi:10.3389/fcvm.2026.1811901)
Supplement: Supplementary file 1 [file Datasheet1.docx]

**Supplementary material**

Supplementary Figure 1. “Sandwich” – like catheter stabilization

Supplementary Figure 2. Farawave 31-mm footprint

Supplementary Figure 3. Esophagus after PFA – outer layer (left) and inner layer (right). Note white spots on the inner layer.

Supplementary Figure 4. Pulmonary trunk after PFA.

Supplementary Figure 5. Aortic root after PFA.
